# Miscellaneous Notices

**Published:** 1854-01

**Authors:** 


### MISCELLANEOUS NOTICES.

*Dr. F. H. Badger.*—We have recently had the pleasure of making the acquaintance of Dr. F. H. Badger, of New Orleans, who for thirty years has sustained a justly deserved high reputation as a dental practitioner. In the operation of filling teeth, to which he has more particularly devoted his attention, he ranks among the first dentists in the union, and, during his visit to Baltimore, which was protracted to four or five weeks, we had frequent opportunities of interchanging views with him upon various subjects connected with operative dentistry. Thus, our personal intercourse with Dr. B. was not only a source of great pleasure, but we obtained from him some valuable practical suggestions which our own experience and the other sources of information of which we had hitherto been able to avail ourselves, had failed to supply. Among other things, he described his method of procedure in filling roots of teeth, and as this is an operation which is attracting considerable attention at this time, we will endeavor to repeat it.

The pulp of the tooth, if remaining, is first destroyed with arsenious acid and creosote, applied on the inside of a small cap made of sheet lead, with a small perforation in the centre, through which the sharp end of a small wire projecting from the end of a canula or tube is passed, and by means of which, it is placed directly upon the exposed pulp. The tube is now moved on the wire until the end comes in contact with the convex part of the cap, when the stylet or wire is withdrawn and both removed without displacing or moving from its proper adjustment the leaden cap. This done, the external opening in the crown of the tooth is filled in the usual way with softened wax, and the application permitted to remain sufficiently long to destroy the vitality of the entire pulp.

The wax and cap containing the arsenious acid, having been taken from the tooth and the pulp removed to the extremity of the root, the external opening, if necessary, is now enlarged and the central chamber in the tooth made as easy of access as possible. In short, the tooth being properly prepared for the reception of the filling, a piece of gold foil, of about two inches in length, and a quarter or a half inch in width at one end and coming to a point at the other, is rolled into as a solid wire as possible. The length of the canal in the root is now ascertained, and if the opening at its extremity is large enough to permit the passage of the point of the wire, the latter is clipped off with a pair of scissors. Several of these sharp cylinders or wires made of foil in the manner as just described, should be prepared previously to commencing the operation of filling, and being thus provided with them, the canal in the root is made dry, by introducing a slender pointed probe made of whalebone, silver or untempered steel, having a few straight fibres of raw cotton wound around it. When the moisture has been completely absorbed, the large end of one of the cylinders is grasped with a pair of properly shaped tweezers. With this instrument the small end is introduced into the canal of the root, and is readily carried up to the extremity of the root. This done, a slim probe, tapering to a point, made of whalebone, or untempered steel, is forced up between the gold and the wall of one side of the canal of the root, firmly compressing the cylinder. The probe is then withdrawn and another and another cylinder introduced, each one in like manner compressed, the canal in the root becoming shorter and shorter, until it is completely filled.

Having filled the root, the remainder of the operation is divided into two parts. The first, consists in filling the central chamber in the crown, and the second and last, in filling the external opening.

*A Lower Molar Tooth with four Roots.*—The senior editor received a letter a few weeks since, enclosing a lower molar with four well developed roots. He has mislaid the letter, and is consequently unable to mention the name of the writer. We would be glad to hear from him again.

*Crystallized Gold.*—Dr. Watts, of Utica, New York, presented to the senior editor, a few weeks since, some beautiful samples of crystallized gold, of his own preparation. He at the same time showed him a ring which he wore on his finger, made from it by Dr. Dwinelle, of Cazenovia, N. Y. It had the appearance of having been formed from a solid piece of metal, it being free from cracks or flaws. The senior editor has filled several teeth with Dr. Watts' preparation of gold, and certainly it realizes his most sanguine expectations. It may be made as solid and as impermeable to the fluids of the mouth as a filling of the best foil, since the crystals when brought in contact with each other by pressure, weld and become a solid mass. It answers in many cases where the tooth is broken away, rendering it necessary to build the gold up above the walls of the cavity a much better purpose. Still it can never be made to supersede the use of gold foil. This can be introduced in most cases with greater facility, and in some cases where it would be impossible to make a good filling with crystallized gold, unless some better method than any at present known, of introducing it, shall be discovered. But so important and valuable does he regard this preparation of gold, that he would not, on any account, dispense, wholly, with the use of it in his practice.

Such of the readers of the Journal as may desire to give Dr. Watts' preparation of gold a trial, will ascertain where it may be procured by referring to his advertisement in the advertising sheet of the present number.

*Cholera.*—A very remarkable statement has been recently made in a communication from Dr. Bury, read to the Medical Society of London. It is to the effect that metallic copper externally applied, in the form of a band, actually arrested the cramps. The communication goes on to say that its author had discovered, from an investigation of about 300,000 cases, that workmen in copper and in steel enjoyed a remarkable exemption from this terrible scourge. The author even pushes his assertion so far as to declare that *removal to a copper mine* had checked choleraic symptoms. The latter statement borders so closely on the marvellous that we may be excused for asking for ample proof before we believe it.

*Mustaches.*—Punch has lately been making himself merry over what he calls the moustachio movement. He pictures terrible looking fellows "bearded like the pard" haunting the railway stations, demanding baggage, and terrifying old ladies who fancy they are beset by banditti.

On the other hand, some medical men are advocating the return to the natural use of the beard, thinking probably that the Almighty knew quite as well as we do where it is best that hair should grow. The hint is thrown out that consumptive patients may be benefitted by the use of mustaches. The Scottish stone-masons have allowed their beards to grow upon their upper lips, so as to obtain a filter which arrests the dust before it can penetrate the lungs. There can be no doubt that a good bushy beard will act in this manner, and so save the air passages no inconsiderable irritation.

Meanwhile it is surprising what a tremendous prejudice exists in some minds against this simple obedience to the dictates of nature. There are still sections of our country in which a man would be ruined who should undertake to turn out a pair of mustaches. We once heard a very worthy but weak old gentleman furiously denouncing certain young men, of whom he knew nothing except that they suffered all their hair to grow. He regarded their mustaches as incontestable evidence of atheistical sentiments.

For ourselves we are obliged to divide the luxury among us, so that our three conjoint faces would present the original distribution of hair, before razors interfered with the plan of nature.

*Artificial Diamonds.*—It will be remembered by our readers who have watched the progress of chemistry for the last thirty years, that much interest was awakened and much discussion excited by the attempt, some years ago, to crystallize carbon by the galvanic battery, or, in other words, to produce diamonds by artificial means. We remember to have seen the venerable Professor Silliman exhibit to his class certain brilliant little particles which had been obtained in this way, from charcoal. Much was said about these at the time, one party contending that they were genuine diamonds, the other that they were only particles of fused silex, originating in the inorganic matter of the coal.

This old discussion bids fair to be revived. We see that M. Despretz has claimed to have effected this modification of carbon by his galvanic battery, under circumstances which do not admit of the hypothesis of fused silex. He placed at the inferior pole of a voltaic battery a cylinder of charcoal, the purity of which was ensured by preparing it from crystallized white sugar candy, and at the superior pole a bundle of fine platinum wires. These elements were so arranged that when the electric light was formed, the charcoal was in the red and the platinum in the violet portion of the arc. After the action was over, he found the carbon collected in a changed form upon the platinum wires. The current was continued for a month, and the powder was found hard enough to polish rubies with great rapidity. When burned it left no residue whatever.

M. Despretz remarks as follows upon these phenomena:

"Have I obtained crystals of carbon which I can separate and weigh, in which I can determine the index of refraction and the angle of polarization without doubt? No; I have simply produced by the electric arc, and by weak voltaic currents, carbon crystallized in black octahedra, in colorless and translucent octahedra, in planes also colorless and translucent, which possess the hardness of the powder of the diamond."

A similar result has been obtained by decomposing a mixture of chloride of carbon and alcohol by weak galvanic currents. The black powder deposited was found to possess equal hardness with that which was sublimed, and rubies was readily polished by it.

*Clerical Gullibility.*—We wonder if there ever was a humbug too transparent, a quackery too contemptible, a dogma too monstrous, to fail in obtaining clerical support. Sherman's lozenges, Jayne's expectorant, Swaim's panacea, Delaney's Indian vegetable blood purifier, homeopathy, hydropathy, Thompsonianism, kinesipathy, physopathy, every thing monstrous, incredible, preposterous and nonsensical isc arefully cherished by some one or other of this white-cravated mob.

It is to no purpose that the medical profession with a liberality that, we are proud to say, is inseparable from the art, attends these gentlemen gratuitously, and showers upon them profusely all the benefits of the hoarded knowledge of centuries. Any shoemaker fresh from the last, ignorant to the last degree, scarcely a perceptible remove from the chimpanzee, can win the parson's confidence and secure his influence. It is disgusting to see these gullible gentry, who know less about the interior of the human body than they do of the geology of the north pole, running about among the mangy members of their flocks, shaking their confidence in their regular medical advisers, and recommending as infallible panaceas, the pills of brother Snob, or the tar-water of sister Snooks. Such a nuisance as this ought to be abated as speedily as possible.

If only some professorship could be established from which these fathers in Israel could be taught the profundity of their own ignorance in all matters pertaining to the physical welfare of their flocks, and the moral obliquity of their conduct in fostering the miserable, murderous, long-eared army of quacks, who eat up the substance and steal the lives of the people, an incalculable benefit would be conferred upon society. Or if it were required of every clergyman that he should get a general idea of medicine, or conscientiously refrain from meddling with what he does not and cannot by any possibility understand, much money and many lives would be saved.

We wish to be distinctly understood. We do not make these charges against the clergy in the main, but only against a portion of them, ignorant, presuming, pretending grannies, who are always meddling in things which do not concern them, and in which their ignorance is like space, infinite, unfathomable. We speak, because we have seen our brethren in the profession suffer from these pretenders and we aim our shafts at them, the patrons of cancer-doctors, and water-doctors, and quack-doctors, the self-constituted keepers of more asses than Abraham ever owned. Many of the clergy, perhaps a majority of them, are too honest and too well informed to encourage quackery in any shape, but it must be confessed that the name of the class we denounce is legion.

The influence of these people being great, the mischief they do is equally extensive. They have, on account of their position, a reputation for intelligence which they do not deserve. Their opinions upon physical science are considered oracular by their followers, and yet on this matter they are, as we have already said, unspeakably ignorant. Any of our readers who has ever heard them promulgate their crude notions about mesmerism, biology, electricity, &c. will bear us out in this assertion.

These remarks have been called out by a parsonic effusion on table-turning, copied into the Dublin Medical Press. The individual who has thus kindly condescended to enlighten the ignorance of a benighted world is, it appears a vicar.

The reverend gentleman was in a party of several gentlemen and ladies, who instituted the common series of scientific experiments upon a table. He is as particular in his accounts of the movements of the table, as a military writer in his description of the evolutions of troops before some decisive battle. He tells how the table lifted up one leg, how it tapped three times on the floor, how it told him what o'clock it was, and what were the ages of the different persons in the room, with a variety of such useful information, which could have been obtained with far less trouble in another way. It is a little amusing to notice the apologies of the reverend gentleman for some of the undeniable mistakes of the table. It sometimes missed the correct age of members of the party, then the vicar gravely tells it was owing to some confusion in the minds of those who catechised the furniture.

This wonderful performance was wound up by a hornpipe from the table. This is not so unparalleled. The same phenomena has repeatedly occurred to several of our friends, after a protracted dinner-party.

Truly spake the old dramatist,

> **"Hood an ass with reverend purple,**
> **And he shall pass for a Cathedral doctor."**

